# Efficacy and Safety of a Balanced Gelatine Solution for Fluid Resuscitation in Sepsis: A Prospective, Randomised, Controlled, Double-Blind Trial-GENIUS Trial

**DOI:** 10.3390/jcm14155323

**Published:** 2025-07-28

**Authors:** Gernot Marx, Jan Benes, Ricard Ferrer, Dietmar Fries, Johannes Ehler, Rolf Dembinski, Peter Rosenberger, Kai Zacharowski, Manuel Sanchez, Karim Asehnoune, Bernd Bachmann-Mennenga, Carole Ichai, Tim-Philipp Simon

**Affiliations:** 1 Department of Intensive Care and Intermediate Care, University Hospital RWTH Aachen, Pauwelsstraße 30, 52074 Aachen, Germany; tsimon@ukaachen.de; 2 Faculty of Medicine in Hradec Kralove, Charles University in Prague, University Hospital Hradec Kralove, Department of Anaesthesiology, Perioperative Medicine and Intensive Care, Masaryk Hospital, J.E. Purkinje University, 400 96 Usti nad Labem, Czech Republic; jan.benes@kzcr.eu; 3 Servicio de Medicina Intensiva, Hospital Universitari Vall d’Hebron, Passeig Vall d’Hebron, 119-129, 08035 Barcelona, Spain; r.ferrer@vhebron.net; 4 Department of Anaesthesia and Critical Care Medicine, Medical University Innsbruck, Anichstraße 35, 6020 Innsbruck, Austria; dietmar.fries@i-med.ac.at; 5 Department of Anaesthesiology and Intensive Care Medicine, Jena University Hospital, Am Klinikum 1, 07747 Jena, Germany; johannes.ehler@med.uni-jena.de; 6 Department of Anaesthesiology and Intensive Care Medicine, University Medical Center Rostock, Schillingallee 35, 18057 Rostock, Germany; 7 Department of Critical Care and Emergency Medicine, Klinikum Bremen-Mitte, St. Jürgen-Straße 1, 28177 Bremen, Germany; rolf.dembinski@klinikum-bremen-mitte.de; 8 Department of Anaesthesiology and Intensive Care Medicine, University Hospital Tübingen, Hoppe-Seyler-Straße 3, 72076 Tübingen, Germany; peter.rosenberger@med.uni-tuebingen.de; 9 Department of Anaesthesiology, Intensive Care Medicine & Pain Therapy, University Hospital Frankfurt, Goethe University, Theodor-Stern-Kai 7, 60590 Frankfurt am Main, Germany; kai.zacharowski@kgu.de; 10 Hospital Universitario La Paz, Instituto de investigación IdiPAZ, Servicio de Medicina Intensiva, Paseo de la Castellana, 261, 28046 Madrid, Spain; manuelsanchezsa@gmail.com; 11 Service d’Anesthésie Réanimation, Nantes Université, Centre Hospitalier Universitaire de Nantes, Pôle Anaesthésie Réanimations, Service d’Anesthésie Réanimation, Chirurgicale 1, Place Alexis Ricordeau, 44093 Nantes, France; karim.asehnoune@chu-nantes.fr; 12 Institute for Anaesthesiology, Intensive Care and Emergency Medicine, Johannes Wesling Hospital Minden, Hans-Nolte-Str. 1, 32429 Minden, Germany; bernd.bachmann-mennenga@muehlenkreiskliniken.de; 13 Service de Réanimation Polyvalente, IRCAN, Inserm U1081, CNRS UMR7284 et CHU de Nice, Hôpital Pasteur 2, 30 Voie Romaine, CHU de Nice, 06000 Nice, France; carole.ichai@unice.fr

**Keywords:** sepsis, fluid resuscitation, gelatine, crystalloid, fluid balance

## Abstract

**Background/Objective**: Sepsis is a leading cause of death in noncoronary intensive care units (ICUs). Fluids for intravascular resuscitation include crystalloids and colloids. There is extensive clinical evidence on colloid use, but large trials comparing gelatine with crystalloid regimens in ICU and septic patients are lacking. This study aimed to determine whether early, protocol-driven volume resuscitation using a gelatine-based regimen achieves hemodynamic stability (HDS) more rapidly than a crystalloid-based regimen in septic patients. **Methods**: This prospective, controlled, randomised, double-blind, multinational phase IV study compared two parallel groups of septic patients receiving a gelatine-based regimen (Gelaspan^®^ 4% and Sterofundin^®^ ISO, B. Braun Melsungen AG each, at a 1:1 ratio) or a crystalloid regimen (Sterofundin^®^ ISO). Primary endpoint was time to first HDS within 48 h after randomisation. Secondary endpoints included fluid overload, fluid balance, and patient outcomes. **Results**: 167 patients were randomised. HDS was achieved after 4.7 h in the gelatine group and after 5.8 h in the crystalloid group (*p* = 0.3716). The gelatine group had a more favourable fluid balance at 24 h (medians: 3463.00 mL vs. 4164.00 mL; *p* = 0.0395) and less fluid overload (medians: 4296.05 vs. 5218.75%; *p* = 0.0217). No differences were observed in serious adverse events or mortality. **Conclusions**: The study provided clinical evidence of balanced gelatine solution for volume resuscitation in septic patients, although it was terminated prematurely. The early and protocol-based administration of gelatine was safe and effective in the enrolled patient population. Time to HDS was not different between groups but the gelatine-based regimen led to better fluid balance and less fluid overload.

## 1. Introduction

Sepsis is a systemic inflammatory syndrome with high incidence, and a major cause of death in intensive care units (ICUs) [1]. Sepsis causes hypovolemia due to capillary leakage and loss of vascular resistance resulting in a reduction in cardiac output, tissue hypoperfusion, and tissue hypoxia and finally in organ dysfunction. Consequently, it is essential in septic patients to compensate for intravascular fluid deficits as quickly as possible [2,3] to achieve haemodynamic stability (HDS), to establish stable blood pressure, and to improve cardiac output and tissue perfusion [4]. Fluid resuscitation needs to be target-controlled to minimise the risk of fluid overload, which is associated with worse outcomes and increased mortality [5,6]. Thus, fluids should be considered as drugs with special indications and contraindications [7]. Guidance on fluid therapy during the four phases of resuscitation (rescue, optimisation, stabilisation, and de-escalation) [7] may pave the way towards precise therapy. This concept contrasts with methods used in earlier larger studies [8], in which patients may have received resuscitation with a higher-than-needed fluid volume, leading to negative side effects [7].

Passive leg raised (PLR)-induced cardiac output/stroke volume (CO/SV) changes are excellent predictors of volume responsiveness with both specificity and sensitivity values within 90–100% [9]. Compared to standard care, dynamic assessments such as PLR, recently recommended by the Surviving Sepsis Campaign [10], may enhance fluid management and improve clinical outcomes [11]. Several types of resuscitation fluids are available. Colloid solutions like albumin, gelatine, and hydroxyethyl starch (HES) may be beneficial for resuscitation for different reasons, including the shorter time and smaller volume needed to achieve HDS, as well as the potential for a longer duration of effect than with crystalloids [12]. HES, however, is contraindicated in septic patients and was suspended in Europe because of its unfavourable risk–benefit balance [13]. Thus, gelatine is commonly used as an alternative colloid solution to treat hypovolaemia in the perioperative area. A balanced gelatine solution was shown to reduce acid–base imbalances when used in the perioperative care of surgical patients [14] (Quelle). However, few trials have been conducted with gelatine in septic patients [15,16,17], and uncertainty regarding its benefit in sepsis persists; thus, the efficacy and safety of modern succinylated gelatine solutions for the treatment of hypovolemia and achieving HDS, as a surrogate for cardiovascular performance, are of major interest. Therefore, the present study aimed to investigate whether HDS in septic patients can be achieved faster with an early and protocol-based approach to volume resuscitation using a gelatine-based regimen in comparison to a crystalloid regimen. Secondary aim was to assess safety and further efficacy of the applied fluid regimens, like fluid balance and patient-related outcomes.

## 2. Materials and Methods

The “gelatine use in ICU and sepsis (GENIUS) study” was a prospective, controlled, randomised, double-blind, multicentre phase IV study with parallel group design conducted at 12 European ICUs. The departments of health of the governments and the ethic committees to which these hospitals are affiliated approved the study protocol. In this emergency setting, a deferred consent procedure was used, and accordingly, patients or their legal representatives provided written informed consent. The study was conducted between 11 April 2016 and 8 December 2021, in compliance with the International Council for Harmonization (ICH) Good Clinical Practice guideline (ICH E6) and in accordance with the ethical standards laid out in the 1964 Declaration of Helsinki and its amendments.

The trial has been registered within the European clinical trial database ‘EudraCT’ (2015-000057-20) and ‘the ClinicalTrials.gov database (NCT02715466). The concepts for design and study rationale have been previously described [18]. The reporting of this study complies with the CONSORT guidelines for clinical trials [19].

An independent data safety monitoring board (DSMB) was established to periodically review in a blinded manner the progress, safety, and critical efficacy variables in accordance with the DSMB charter and a separate statistical analysis plan (SAP). The DSMB consisted of two clinical experts and one statistician, who were not involved in study conduct.

### 2.1. Participants

Adult patients (≥18 years) diagnosed with severe sepsis/septic shock at ICU admission or during the ICU stay based on the American College of Chest Physicians/Society of Critical Care Medicine (ACCP/SCCM) criteria were eligible. Patients had to be on ongoing antibiotic therapy (started prior to randomisation) and fluid responsive (defined as an increase of >10% in mean arterial pressure (MAP) after passive leg raising (PLR) or fluid challenge of a maximum of 250 mL crystalloid solution), with a body weight below 140 kg. Patients were planned to be enrolled within 90 min after diagnosis of severe sepsis/septic shock at ICU admission or during the ICU stay. The following additional inclusion criteria applied: negative pregnancy test and signed informed consent/deferred consent.

Reasons for exclusion were the administration of HES, dextran solutions, or >500 mL of gelatine solutions within 24 h prior to randomisation; death expected within the next 48 h (moribund patients as defined by American Society of Anesthesiologists (ASA) ≥ class V); expected need for pressure infusions; confirmed acute SARS-CoV-2 (COVID-19) infection; a requirement for renal support; renal failure; severe congestive cardiac dysfunction; therapeutic heparin medication due to chronic coagulation disease/anticoagulation medication (i.e., partial thromboplastin time > 60 s); acute burn injuries; severe general oedema; hypersensitivity to the active substance or ingredients of the IMPs; hypersensitivity to galactose-alpha-1,3-galactose (alpha-Gal) or known allergy to red meat (mammalian meat) and offal; hypervolaemia/hyperhydration; hyperkalaemia; hypercalcaemia; metabolic alkalosis; or simultaneous participation in another interventional clinical trial. Inclusion and exclusion criteria are listed in Supplementary S1.

All ITT (intention to treat) patients who received at least one dose of investigational medicinal product (IMP) were included in the safety analysis set (SAF).

### 2.2. Randomisation, Blinding, Unblinding

The list of treatment assignments considering the stratification for site and red blood cell (RBC) pretreatment was generated by an independent statistician who was not involved in the final data analyses. The randomisation list was generated prior study initiation using random permuted blocks of size 4. Investigational medicinal test and reference products (IMP) were blinded. Blinding of IMP was performed in advance by the sponsor as a part of the sample manufacturing process. Treatment allocation was concealed using prerandomised and covered bottles as well as coloured infusion lines. The allocation to treatment groups was not known to investigators or other persons involved in the study. Except for emergency reasons, the study was only unblinded after the closure of the database and determination of the analysis populations in a blind data review meeting.

### 2.3. Intervention

Patients assigned to the gelatine group received one bottle of crystalloids (i.e., 1 × 500 mL Sterofundin^®^ ISO) for each bottle of administered gelatine (i.e., 1 × 500 mL Gelaspan^®^ 4%) according to routine medical practice [2]. Patients in the crystalloid group received Sterofundin^®^ ISO only. Treatment with IMP followed a protocol-based approach as previously described [18] and was given until achievement of confirmed HDS, up to the maximum daily dose of 30 mL.kg^−1^, or until 48 h after randomisation, whichever occurred first. Fluid requirements exceeding the daily maximum dose of 30 mL.kg^−1^ for the IMPs were facilitated with the use of crystalloids only. Volume responsiveness and IMP administration during the treatment phase were continuously assessed via the PLR manoeuvre and MAP change or fluid challenges and SVI or MAP change depending on the use of a haemodynamic monitoring system to avoid fluid overload. The PLR test consisted of measuring the hemodynamic effects of a leg elevation up to 45°. The PLR test was performed before randomisation and after administration of a maximum of two bottles of IMPs (i.e., 1000 mL) during the first treatment phase. During treatment with fluids, MAP was continuously titrated to a value greater than 65 mmHg with norepinephrine.

If the patient was no longer volume responsive, administration of study fluids was discontinued and criteria for HDS were reassessed. Inotropic therapy was administered if HDS was not established. The administration of study medication continued if the patient was volume responsive again (tested via PLR). To assess safety and efficacy, patients were examined daily starting 48 h after randomisation until Day 28 or ICU discharge, whichever occurred first. Follow-up visits/calls (FU 1 and FU 2) were scheduled on Day 28 and Day 90 after randomisation [18].

### 2.4. Outcome Measures

The primary study objective was to investigate the efficacy of early target-controlled fluid resuscitation using a gelatine-based regimen compared to a crystalloid regimen in achieving HDS in severe sepsis/septic shock patients with hypovolaemia. The primary endpoint was the time needed to achieve the first HDS. This was assessed by measuring the time elapsed between the start of IMP administration and first/initial HDS. HDS was defined as MAP > 65 mmHg, and fulfilment of two of the following criteria: (1) arterial lactate decrease > 10% within the last 6 h or lactate < 2.4 mmol.L^−1^, (2) urine production > 0.5 mL.kg^−1^.h^−1^, and (3) central venous oxygen saturation (ScvO2) > 70% for at least 4 h. The fulfilment of the HDS criteria had to be confirmed after at least 2 h and 4 h to assess the patient as hemodynamically stable; otherwise, study treatment according to the volume algorithm was to be resumed.

The secondary endpoints were safety and further efficacy of the applied fluid regimens, like fluid balance, fluid overload, and patient-related outcomes (for a full list, see Supplementary S2).

Fluid balance [mL] was calculated as “Fluid Input [mL] − Fluid Output [mL]”.

Fluid overload [%] was calculated as “[(Fluid Intake − Fluid Output)/Weight] × 100”.

### 2.5. Statistical Methods

Sample size calculation has been previously described [18] and was based on data from a previous clinical trial [20]. The sample size was calculated as 253 patients per group, based on an effect size of 0.25, α = 5% (two-sided), and 80% power. Allowing for a 20% dropout rate, the final sample size was set at 304 per group. Due to premature study termination, the target sample size of 608 patients was not reached.

After database closure and de-blinding of the study, an independent Contract Research Organisation performed statistical analysis according to the predefined SAP. The SAP was finalised after study protocol publication and can be accessed via clinicaltrials.gov.

SOFA renal score 0–4 was categorised to level 1–5 due to data processing.

Calculations of creatinine clearance (CCr) were performed using the Cockcroft-Gault formula and SCr-based eGFR using the CKD-EPI (SCr) equation as defined in SAP.

Based on the study design, data were analysed using an intention-to-treat approach (ITT) for the primary analyses. All statistical tests for group comparisons were two-sided with a significance level of 5%. This also applied for predefined subgroup analyses (Supplementary S3). These and all tests of secondary variables were considered exploratory data analyses, and no adjustments for multiple testing have been made. Missing values were not imputed except partial dates and times. Data were analysed using SAS version 9.4.

## 3. Results

### 3.1. Patient Characteristics

**Figure 1:**
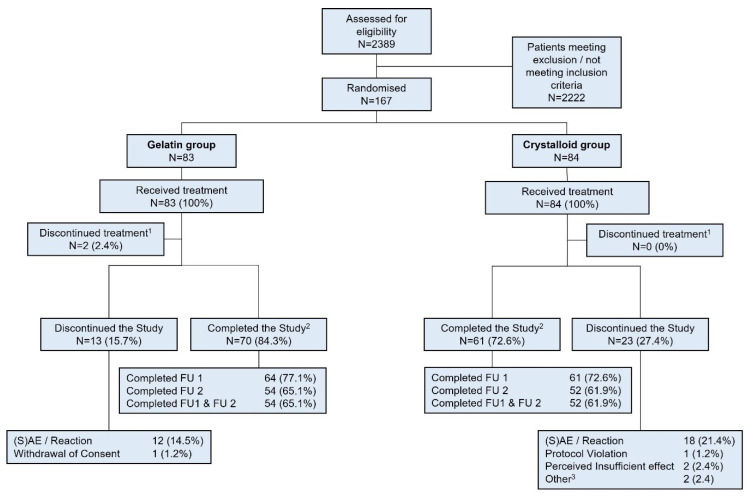
Study flowchart. The CONSORT flowchart shows enrolment, randomisation, and follow-up. Patients were excluded if they ^1^ prematurely discontinued the study treatment due to adverse events, ^2^ completed the study from randomisation to Day 28 or ICU discharge, whichever occurred first, or ^3^ were transferred to the ICU of an external hospital or received emergency surgery due to the initial disease.

Between 11 April 2016 and 18 March 2021, 167 patients with severe sepsis/septic shock were randomised until premature study termination. In total, 83 patients were assigned to receive the gelatine-based regimen and 84 patients were assigned to receive the crystalloid regimen. A total of 131 patients completed the study (Figure 1).

Patient and baseline disease characteristics were balanced in both groups, but mostly surgical patients were enrolled (65.3%) and the proportion of patients diagnosed with septic shock was higher than that of patients with severe sepsis (77.2% and 22.8%, respectively) (Table 1).

### 3.2. Primary Outcome: Time to First Achieved HDS

**Figure 2:**
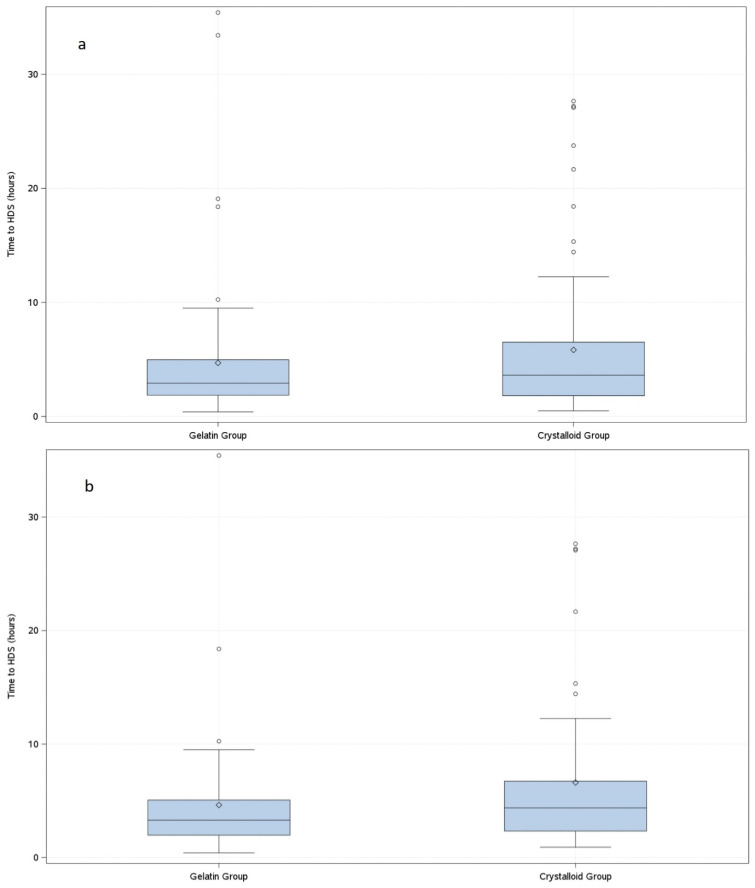
Box plot of time to haemodynamic stability (ITT). The length of the box represents the interquartile range (IQR). The horizontal line in the box interior represents the group median. The symbol in the box interior represents the group mean. The whiskers extend to 1.5 times the IQR above the upper quartile and below the lower quartile. Circles above whiskers represent outliers. (**a**) Box plot of the time to haemodynamic stability of all patients (ITT); (**b**) Predefined subgroup analysis of the primary efficacy endpoint in surgical patients: box plot of time to haemodynamic stability of surgical patients (ITT).

Of 167 patients, 77 (92.8%) patients in the gelatine group and 74 (88.1%) patients in the crystalloid group achieved HDS, confirmed 2 h and 4 h after the achievement of the first HDS. In the gelatine group, HDS was achieved after 4.7 h and in the crystalloid group after 5.8 h (*p* = 0.3716) (Figure 2).

In the predefined subgroup of surgical patients, time to HDS was 4.6 h following treatment with gelatine, compared to 6.6 h after crystalloid administration (*p* = 0.0298, stratified by site and RBC pretreatment) (Figure 2). HDS data of all further subgroup analyses are presented in Supplementary Table S1.

The use of vasopressors and inotropes was similar between treatment groups.

In total, 58 (69.9%) patients in the gelatine group and 55 (65.5%) patients in the crystalloid group had a least one concomitant vasopressor treatment, while 19 patients (22.9%) in the gelatine group and 18 patients (21.4%) in the crystalloid group were treated at least once with one inotropic medication. Amongst others, norepinephrine was given in 58 patients (69.9%) in the gelatine group and 53 patients (63.1%) in the crystalloid group. The treatment with inotropic agents included mainly dobutamine (14 patients (16.8%) in the gelatine group and 12 patients (14.3%) in the crystalloid group). Further details are given in Supplementary Table S2.

### 3.3. Fluid Intake/Fluid Balance

In the gelatine group, median fluid intake, median fluid balance, and median fluid overload were lower during the first 24 h of treatment compared to the crystalloid group (Table 2). There were significant differences in fluid balance (median: 3463.00 mL vs. 4164.00 mL; *p* = 0.0395) and fluid overload (4296.05% vs. 5218.75%; *p* = 0.0217). No notable differences between groups were detected after 48 h.

### 3.4. Patient-Related Outcome Variables

Patient-related outcome variables were comparable between treatment groups (Supplementary Table S2).

Among the safety assessments, renal function was evaluated. The DSMB advised to terminate the study prematurely due to their observation of increased SCr values in patients with preexisting renal impairment, i.e., patients with SOFA renal baseline scores of 2–4. The final analysis of this predefined subgroup, however, revealed a trend towards decreasing mean SCr over time in both study groups (Supplementary Table S4), but SCr decreased slower in the gelatine group. For the treatment groups with SOFA renal baseline scores of 0–1, however, there was no difference (Supplementary Table S4). No significant changes were observed in the Kidney Disease Improving Global Outcome (KDIGO) score (SCr-based calculated) over the 7-day assessment period after randomisation (Supplementary Table S3).

**Figure 3:**
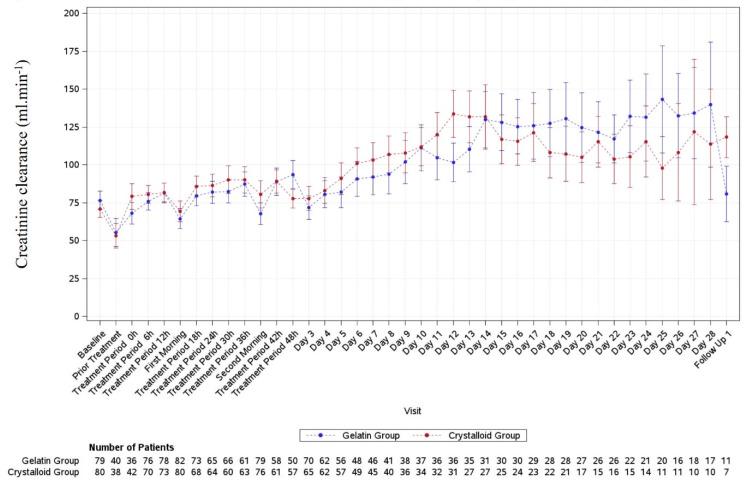
Line plot of creatinine clearance (CCr) over time (until Day 28/ICU discharge/study discontinuation) by treatment group. Furthermore, data from FU1 are shown. Mean values +/− standard errors are presented with dots and bars, respectively.

The calculated mean CCr at baseline was comparable (76.5 mL.min^−1^ in the gelatine group and 70.8 mL.min^−1^ in the crystalloid group) and increased over time in both groups. There were no significant differences between the groups (Figure 3).

The development of mean estimated glomerular filtration rate (eGFR) (SCr-based calculated) over time and urine output was comparable between the treatment groups (Supplementary Table S4). The occurrence of new RRT/kidney disease after study termination (between day 29 and day 90) was identical between treatment groups (n = 3) (Supplementary Table S2).

### 3.5. Adverse Events

Four nonserious adverse reactions were reported in the gelatine group and one in the crystalloid group (Supplementary Table S5). No anaphylactic or anaphylactoid reactions occurred. The overall number of patients with treatment-emergent adverse events (TEAEs) was similar between treatment groups (62 (74.7%) patients in the gelatine group and 61 (72.6%) patients in the crystalloid group). A total of 38.3% of patients in the SAF group experienced at least one serious TEAE, with the number of patients with serious TEAEs being similar between both groups (30 (36.1%) patients in the gelatine group and 34 (40.5%) patients in the crystalloid group) (Supplementary Table S6).

From randomisation to Day 28, 11 patients in the gelatine group and 18 patients died in the crystalloid group. In total, 52 patients died by Day 90 (±10 days), with 26 patients per treatment group (Supplementary Table S7).

## 4. Discussion

The GENIUS study is the first study comparing a balanced succinylated gelatine/crystalloid regimen with a balanced crystalloid regimen in patients diagnosed with severe sepsis or septic shock. In this randomised controlled double-blinded study, the primary endpoint HDS was achieved after 4.7 h (gelatine group) and after 5.8 h (crystalloid group). Even though the study was underpowered due to premature termination, results from predefined subgroup analysis indicated that gelatine may have an advantage in surgical septic patients. Reasons might be a difference of phenotypes and pathophysiology in septic patients by site of infection and aetiology [21,22].

There is still controversy regarding the optimal fluid resuscitation strategy in the early hours of sepsis. Rivers et al. [23] first described the concept of early goal directed therapy (EGDT), which was challenged by three multicentre trials—ARISE [24], ProCESS [25], and ProMISe [26]. In the initial Rivers trial, the EGDT group received 42% more fluids than the control group within the first 6 h, whereas after 72 h, similar amounts of fluids were administered in both groups. In ProCESS, the EGDT group received only 16% more fluids, whereas in ARISE and ProMISe, the difference was small (4.1% and 4.4%, respectively). In all three trials, approximately 45% to 60% of the resuscitation fluids were given before the protocol commenced [27,28]. The results from Corl et al. indicate that both tightly restricting and aggressively administering intravenous fluids in patients with sepsis and septic shock may not be advisable. Instead, moderate intravenous fluid given before the end of day one in hospital was linked to lower mortality [29].

Sakr et al. [30] investigated the influence of early fluid balance on outcome of septic patients and demonstrated that initial fluid therapy seems to be necessary but especially in the early treatment period of 24 h and only up to 3 days.

When applied in the early phase up to 6 h after sepsis diagnosis, colloid solutions can maintain intravascular osmotic pressure, resulting in rapid and lasting circulatory stabilisation of septic shock patients [15,16]. In contrast to previous studies, in which investigational products were still applied several hours after initial HDS [8,31,32], GENIUS study patients were randomised within one hour after diagnosis of sepsis, as documented in the patient chart. This early and immediate treatment, which is in line with the Rivers protocol, may explain the obtained results with balanced gelatine.

In the GENIUS study, fluid resuscitation in septic patients was also guided by a protocol-based fluid algorithm [18] and the study followed evidenced-based parameters for the diagnosis of fluid deficits and assessment of HDS [18], being the first study in line with the German guideline for volume therapy [33]. Determination of fluid deficits and achievement of HDS was based on the PLR manoeuvre or exogenous fluid challenge and appropriate dynamic haemodynamic parameters (MAP, SVI) [9]. The concept of PLR was recently confirmed in a multicentre study [34]. Thus, adequate measurements instead of no longer recommended parameters such as CVP [10,33] were used. Additionally, in contrast to previous trials we followed modern fluid resuscitation concepts using balanced solutions within the test as well as in the control group, as their use results in fewer metabolic derangements, less hyperchloremia, and less metabolic acidosis than normal saline [35].

In the GENIUS study, the observed statistically significant differences in fluid balance and fluid overload suggested an improved intravascular volume stabilisation when using gelatine. This was also demonstrated by Trof et al. [16] and Molnar et al. [15] in patients during early sepsis. Fluid overload increases tissue oxygen tension and is associated with a significantly higher mortality rate [6] and occurrence of adverse events in critical patients [36].

In general, morbidity and mortality from sepsis or septic shock remain high, with recently reported average 30-day and 90-day mortality of 34.7% and 38.5%, respectively [37]. In our study, no significant differences between treatment groups with respect to 28-day and 90-day mortality rates were detected.

In septic patients, renal failure is a frequent complication [38,39] and the use of colloids was reported to worsen renal function [40]. A recent network meta-analysis concluded that gelatine was associated with a lower incidence of acute kidney injury compared to HES but had had worse outcomes in terms of mortality, continuous renal replacement therapy, and hospital stay duration [41]. Due to safety issues during previous studies [31], measures for minimising potential risks during the GENIUS study were undertaken by involving an independent DSMB. The DSMB’s recommendation to terminate the study early was a precautionary measure due to initial observations of varying SCr levels over time between treatment groups. However, this observation was not substantiated. There is the assumption that adverse renal effects of gelatine may be more pronounced in patients with preexisting renal dysfunction [40]. Results showed that SCr values were increased in the subgroup of patients with baseline SOFA renal score 2–4 in both treatment groups, but values decreased over time. In the gelatine group, SCr values decreased slower compared to the crystalloid group. In patients with baseline SOFA renal score 0–1, SCr values remained within the normal range. Calculated mean CCr values over time were comparable. Recently, the importance of CCr for treatment guidance and renal function has been emphasised [42].

Strengths of this study: Despite the premature termination and the resulting underpowered sample size, the GENIUS study provides important value by addressing methodological shortcomings of previous trials. Notably, it is one of the first randomised controlled, double-blind, multicentre, international trial to implement a strictly protocol-based, early fluid resuscitation strategy using balanced gelatine as well as a balanced electrolyte solution in septic patients. Patients were randomised within one hour of sepsis diagnosis, ensuring that the intervention occurred during the critical early phase of circulatory instability—an approach aligned with modern sepsis guidelines [10] but rarely achieved in earlier studies. Furthermore, according to current guidelines, dynamic hemodynamic monitoring (PLR, SVI) instead of static parameters like CVP were used [10,33] These methodological strengths enhance the clinical relevance of the findings, particularly the observed improvements in fluid balance and fluid overload, which are increasingly recognised as key determinants of outcome in sepsis. Furthermore, safety evaluations were comparable for both study groups.

Limitations of this study: The planned sample size was not reached due to premature termination. Scientifically, it was important to investigate the early phase of fluid resuscitation using a protocol-based approach in sepsis. However, enrolment within the predefined timeframe of 90 min after diagnosis of severe sepsis/septic shock was challenging and contributed to slow recruitment. Recruitment was further slowed down by the COVID-19 pandemic and the associated utilisation of ICUs. After study protocol approval and during the recruitment phase, new definitions of sepsis and septic shock were published [43]. However, considering clinical practice at the time of enrolment, the study continued with the former established definitions [44].

## 5. Conclusions

To our knowledge, this is the first randomised controlled, double-blind, multicentric, international trial evaluating gelatine in septic patients using evidence-based PLR for both indication and treatment of hemodynamic instability. The applied early and protocol-based approach showed that fluid therapy can be guided efficiently, although the study was terminated prematurely. Importantly, the results indicate the safety of a regimen using balanced gelatine in combination with balanced crystalloids. In the overall study group, no statistically significant differences were detected in achieving initial hemodynamic stability (HDS). In surgical patients, HDS was achieved faster using the gelatine-based regimen. Compared to the crystalloid regimen, the gelatine-based regimen achieved better fluid balance and less fluid overload, and hence, patients are likely to benefit from earlier HDS while requiring less fluid volume.

## Figures and Tables

**Table 1:** Patient and baseline disease characteristics.

Variable		Gelatine Group N = 83	Crystalloid Group N = 84	Total N = 167
Sex, n (%)	Male	62 (74.7)	64 (76.2)	126 (75.4)
	Female	21 (25.3)	20 (23.8)	41 (24.6)
Age (years)	Mean (SD)	65.3 (14.20)	65.9 (14.14)	65.6 (14.13)
Weight (kg)	Mean (SD)	83.2 (16.44)	80.9 (15.23)	82.1 (15.83)
Disease characteristics				
Type of patient, n (%)	Trauma	8 (9.6)	5 (6.0)	13 (7.8)
	Medical	23 (27.7)	22 (26.2)	45 (26.9)
	Surgical	52 (62.7)	57 (67.9)	109 (65.3)
Diagnosis, n (%)	Severe sepsis	20 (24.1)	18 (21.4)	38 (22.8)
	Septic shock	63 (75.9)	66 (78.6)	129 (77.2)
Time from diagnosis to randomisation or ICU admission to randomisation (min) ^1.^	Mean (SD)	60.4 (28.50)	58.4 (25.98)	59.4 (27.20)
Fluid input 24 h prior to randomisation, n (%)	Yes	77 (92.8)	77 (91.7)	154 (92.2)
	No	6 (7.2)	7 (8.3)	13 (7.8)
Total amount of fluids (mL) 24 h prior to randomisation	n	77	76	153
	Mean (SD)	2309.6 (1504.62)	2439.5 (1802.82)	2374.1 (1655.26)
RBC therapy 24 h prior to randomisation, n (%)	Yes	14 (16.9)	14 (16.7)	28 (16.8)
	No	69 (83.1)	70 (83.3)	139 (83.2)
Total volume (mL) of RBCs 24 h prior to randomisation	n	14	13	27
	Mean (SD)	538.2 (274.46)	727.7 (568.39)	629.4 (442.81)
Lactate prior treatment (mmol.L^−1^)	n	52	48	100
	Mean (SD)	2.6 (2.30)	2.6 (2.64)	2.6 (2.46)
APACHE II total score	n	78	76	154
	Mean (SD)	23.6 (6.54)	22.8 (7.47)	23.2 (7.00)
SOFA total score	n	77	78	155
	Mean (SD)	8.3 (2.73)	8.1 (3.39)	8.2 (3.07)

^1.^ Time from severe sepsis/septic shock diagnosis to randomisation for patients diagnosed during the ICU stay or time from ICU admission to randomisation for patients diagnosed at ICU admission.

**Table 2:** Fluid intake summary (SAF).

Time	Parameter	Median (Q1–Q3) [n]		*p* Value *
		Gelatine Group N = 83	Crystalloid Group N = 84	
0–24 h	Fluid intake (mL)	6046.00 (4246.00–7980.00) [83]	6333.00 (4870.00–8970.00) [81]	0.0712
	Fluid output (mL)	2135.00 (1035.00–3262.00) [83]	1730.00 (1270.00–2680.00) [81]	0.3230
	Fluid balance (mL)	3463.00 (2020.00–5437.00) [83]	4164.00 (2682.00–7526.00) [81]	0.0395
	Fluid overload (%)	4296.05 (2424.28–6946.15) [83]	5218.75 (3180.00–8884.71) [81]	0.0217
24–48 h	Fluid intake (mL)	3379.00 (2439.00–4867.00) [83]	3848.00 (2880.00–5001.00) [78]	0.1491
	Fluid output (mL)	1970.00 (990.00–2810.00) [83]	1990.00 (1290.00–2954.00) [78]	0.5370
	Fluid balance (mL)	1408.00 (−71.00–2696.00) [83]	1733.00 (607.00–2621.00) [78]	0.2214
	Fluid overload (%)	1586.67 (−97.26–3880.00) [83]	2093.25 (642.86–3630.00) [78]	0.2564

* Mann-Whitney U test (two sided) *p* value for comparison of medians in the gelatine group versus the crystalloid group; N = number of patients; n = number of patients with available data; Q1 = first quartile; Q3 = third quartile.

## Data Availability

The datasets used and/or analysed during the current study are available from the corresponding author on reasonable request.

## Supplementary Materials

The following supporting information can be downloaded at: https://www.mdpi.com/article/10.3390/jcm14155323/s1. SDC1_Supplementary S1: In- and Exclusion Criteria.docx; SDC2_Supplementary S2: Secondary Outcomes.docx; SDC3_Supplementary S3: pre-defined subgroups.docx; SDC4_Figure S1: Line Plot of SCr over time.pdf; SDC5_Table S1: Time to Haemodynamic Stability by Subgroups.docx; SDC6_Table S2: Clinical outcome parameter for the overall study group.docx; SDC7_Table S3: Shift table of KDIGO stages over study period.docx; SDC8_Table S4: eGFR and urine output.docx; SDC9_Table S5: Summary of Adverse Reactions.docx; SDC10_Table S6: Treatment-Emergent Serious Adverse Events.docx; SDC11_Table S7: Summary of Deaths.docx.
