# Supplementary material for: Efficacy and Safety of a Balanced Gelatine Solution for Fluid Resuscitation in Sepsis: A Prospective, Randomised, Controlled, Double-Blind Trial-GENIUS Trial
Source: J Clin Med. 2025 Jul 28;14(15):5323. doi: 10.3390/jcm14155323 (PMC12346933; doi:10.3390/jcm14155323)
Supplement: Supplementary file 1 [file jcm-14-05323-s001.zip › SDC10_Table S6_Treatment-Emergent Serious Adverse Events.pdf]

Table S6. Treatment-emergent serious adverse events (TEAE) reported for > 1 patient in either treatment group by primary system organ class and preferred term (SAF). N = total number of patients, N_1 = number of patients with at least one TEAE, N_2 = number of events.

System Organ Class<br>Preferred Term	Gelatine Group<br>N = 83		Crystalloid Group<br>N = 84		Total<br>N = 167	
	N_1 (%)	N_2	N_1 (%)	N_2	N_1 (%)	N_2
Serious TEAEs	30 (36.1)	58	34 (40.5)	76	64 (38.3)	134
Infections and infestations	10 (12.0)	10	12 (14.3)	12	22 (13.2)	22
Septic shock	6 (7.2)	6	9 (10.7)	9	15 (9.0)	15
Abdominal abscess	0	0	2 (2.4)	2	2 (1.2)	2
Pneumonia	2 (2.4)	2	0	0	2 (1.2)	2
General disorders and administration site conditions	7 (8.4)	7	12 (14.3)	12	19 (11.4)	19
Multiple organ dysfunction syndrome	7 (8.4)	7	12 (14.3)	12	19 (11.4)	19
Respiratory, thoracic, and mediastinal disorders	7 (8.4)	7	9 (10.7)	9	16 (9.6)	16
Respiratory failure	2 (2.4)	2	4 (4.8)	4	6 (3.6)	6
Cardiac disorders	5 (6.0)	6	8 (9.5)	11	13 (7.8)	17
Cardiac arrest	0	0	4 (4.8)	5	4 (2.4)	5
Atrial fibrillation	2 (2.4)	2	1 (1.2)	1	3 (1.8)	3
Ventricular tachycardia	0	0	3 (3.6)	3	3 (1.8)	3
Injury, poisoning and procedural complications	5 (6.0)	5	6 (7.1)	7	11 (6.6)	12
Failure to anastomose	3 (3.6)	3	3 (3.6)	3	6 (3.6)	6
Gastrointestinal disorders	5 (6.0)	5	6 (7.1)	6	11 (6.6)	11
Intestinal ischaemia	2 (2.4)	2	1 (1.2)	1	3 (1.8)	3
Vascular disorders	5 (6.0)	7	5 (6.0)	6	10 (6.0)	13
Hypotension	2 (2.4)	2	2 (2.4)	2	4 (2.4)	4
Nervous system disorders	5 (6.0)	5	5 (6.0)	6	10 (6.0)	11
Brain injury	0	0	2 (2.4)	2	2 (1.2)	2
Blood and lymphatic system disorders	1 (1.2)	1	2 (2.4)	2	3 (1.8)	3
Hepatobiliary disorders	1 (1.2)	1	2 (2.4)	2	3 (1.8)	3
Renal and urinary disorders	2 (2.4)	2	1 (1.2)	1	3 (1.8)	3
Acute kidney injury	2 (2.4)	2	1 (1.2)	1	3 (1.8)	3
