# Supplementary material for: Efficacy and Safety of a Balanced Gelatine Solution for Fluid Resuscitation in Sepsis: A Prospective, Randomised, Controlled, Double-Blind Trial-GENIUS Trial
Source: J Clin Med. 2025 Jul 28;14(15):5323. doi: 10.3390/jcm14155323 (PMC12346933; doi:10.3390/jcm14155323)
Supplement: Supplementary file 1 [file jcm-14-05323-s001.zip › SDC11_Table S7_Summary of Deaths.pdf]

Table S7. Summary of deaths. ARD = acute respiratory distress; FU = Follow up; ICU = intensive care unit; N = total number of patients.

**Study Period**<br>**Reported Cause of Death**	**Number (%) of Patients**		
	**Gelatine group N = 83**	**Crystalloid Group, N = 84**	**Total N = 167**
**Death during study period (due to AE)**	**11 (13.3)**	**18 (21.4)**	**29 (17.4)**
**Death during FU period**	**15 (18.1)**	**8 (9.5)**	**23 (13.8)**
- From ICU discharge to Day 28 (FU 1)	10 (12.0)	3 (3.6)	13 (7.8)
- After Day 28 (FU 2)	5 (6.0)	5 (6.0)	10 (6.0)
