# Supplementary material for: Efficacy and Safety of a Balanced Gelatine Solution for Fluid Resuscitation in Sepsis: A Prospective, Randomised, Controlled, Double-Blind Trial-GENIUS Trial
Source: J Clin Med. 2025 Jul 28;14(15):5323. doi: 10.3390/jcm14155323 (PMC12346933; doi:10.3390/jcm14155323)
Supplement: Supplementary file 1 [file jcm-14-05323-s001.zip › SDC1_Supplementary S1_In- and Exclusion Criteria.pdf]

Supplementary S1.

Inclusion criteria:

- Male or female patients ≥ 18 years of age
- Women of childbearing potential must test negative on standard pregnancy test (urine or serum)
- Patients with body weight ≤ 140 kg
- Patients diagnosed severe sepsis / septic shock at admission on ICU who can be enrolled within 90 min after admission OR patients diagnosed severe sepsis / septic shock during ICU stay who can be enrolled within 90 min after diagnosis
- Patients where antibiotic therapy has already been started (prior to randomization)
- Patients who are fluid responsive. Fluid responsiveness is defined as increase of > 10% in mean arterial pressure (MAP) after PLR or fluid challenge (max. 250 ml crystalloid solution)
- Signed informed consent by patient, legal representative or authorized person or deferred consent

Exclusion criteria:

Patients meeting one or more of the following criteria could not be enrolled in this clinical study:

- Administration of hydroxyethyl starch (HES), dextran solutions or > 500 mL of gelatine solutions within the 24 h prior to randomization
- Death expected within the next 48 h (moribund patients as defined by ASA ≥ class V)

- Patients with confirmed acute SARS-CoV-2 infection (as available from routine medical records/ patient chart)
- Patients for whom the need of pressure infusions was expected
- Requirement for renal support (either continuous or discontinuous techniques, including intermittent hemodialysis, hemofiltration and hemodiafiltration)
- Patients receiving therapeutic heparin medication due to chronic coagulation disease / anticoagulation medication (i.e., partial thromboplastin time > 60 seconds)
- Acutely burned patients, with burns defined as having any of the following before the administration of IMP: > 10% of BSA classified as 3rd or 2nd-degree not yet re-epithelialised
- Renal failure with oliguria or anuria
- Severe general oedema
- Severe congestive cardiac failure
- Hypersensitivity to the active substance or ingredients of the IMPs
- Hypersensitivity to galactose-α-1,3-galactose (alpha-Gal) or known allergy to red meat (mammal meat) and offal
- Hypervolemia / hyperhydration
- Hyperkalemia
- Hypercalcemia
- Metabolic alkalosis
- Simultaneous participation in another interventional clinical trial (drugs or medical devices studies)
