# Supplementary material for: Efficacy and Safety of a Balanced Gelatine Solution for Fluid Resuscitation in Sepsis: A Prospective, Randomised, Controlled, Double-Blind Trial-GENIUS Trial
Source: J Clin Med. 2025 Jul 28;14(15):5323. doi: 10.3390/jcm14155323 (PMC12346933; doi:10.3390/jcm14155323)
Supplement: Supplementary file 1 [file jcm-14-05323-s001.zip › SDC2_Supplementary S2_Secondary Outcomes.pdf]

Supplementary S2. List of secondary efficacy and safety outcomes.

**Objectives**	**Endpoints**
**Secondary**	**Efficacy**
• Investigation of further efficacy parameters of the applied volume replacement regimes	• Fluid responsiveness upon passive leg raising (PLR) and MAP during the 1st treatment phase and by exogenous fluid challenge (FC) and stroke volume index (SVI) using a haemodynamic monitoring system during the 2nd treatment phase
	• Haemodynamic parameters between randomisation and study end per patient (Day 28/ICU discharge, whatever occurred first), and changes over time compared to baseline
	• Tissue oxygenation and acid base balance / blood gas analysis (BGA) between randomisation and study end per patient (Day 28/ICU discharge whatever occurred first) and changes over time
	• Clinical outcome, including triggers for starting renal replacement therapy (RRT) over time
	• Time to ICU discharge
	• Disease severity evaluation on the Sequential Organ Failure Assessment (SOFA) score, the Simplified Acute Physiology Score II (SAPS II) score, and the Acute Physiology and Chronic Health Evaluation II (APACHE II) score including changes from baseline over time
	• Frequencies of patients with new sepsis/septic shock diagnosis as recorded in the eCRF and as per new severe sepsis/septic shock definition
**Secondary**	**Safety**
• Investigation of safety parameters of the applied volume replacement regimes	• Incidence, severity, seriousness, and causality of treatment-emergent adverse events (TEAEs) and adverse reactions (ARs)
	• Renal function over time (serum creatinine [SCr], urine creatinine, serum blood urea nitrogen [BUN], need/indication of RRT, urine output, creatinine clearance [Ccr], estimated Glomerular Filtration Rate (eGFR), Kidney Disease Improving Global Outcome (KDIGO) score)
	• Clinical laboratory for coagulation over time (prothrombin time (PT), activated partial thromboplastin time (aPTT), international norm ratio (INR), site-specific/optional evaluations of antithrombin (AT), fibrinogen, and platelets absolute)
	• Clinical laboratory for hepatic function over time (bilirubin)
	• Frequency of concomitant medications intake during treatment period: red blood cell (RBC),

	fresh frozen plasma (FFP) and other blood products, norepinephrine therapy, vasopressors, inotropic agents, antibiotic agents, anticoagulant agents, nephrotoxic agents, contrast agents, and crystalloid solutions
	• Vital signs (blood pressure, heart rate, and temperature)
**Other**	**Follow Up**
• Investigation of safety and efficacy including patient's quality of life during the follow-up (FU)	• Colloid therapy from ICU discharge to hospital discharge or Day 28, whatever occurred first
	• Mortality at Day 28 and Day 90, including cause of death
	• Last available SCr data from ICU discharge to hospital discharge or Day 28, whatever occurred first
	• New RRT / kidney disease at Day 90, including cause as applicable
	• Health related quality of life (HRQoL) at Day 90
