# Supplementary material for: Efficacy and Safety of a Balanced Gelatine Solution for Fluid Resuscitation in Sepsis: A Prospective, Randomised, Controlled, Double-Blind Trial-GENIUS Trial
Source: J Clin Med. 2025 Jul 28;14(15):5323. doi: 10.3390/jcm14155323 (PMC12346933; doi:10.3390/jcm14155323)
Supplement: Supplementary file 1 [file jcm-14-05323-s001.zip › SDC3_Supplementary S3_pre-defined subgroups.pdf]

Supplementary S3. Pre-defined subgroup analyses.

The following subgroup categories were defined for analysis of the primary endpoint:

- Country
- Investigational site
- RBC pre-treatment 24 h prior randomisation according to the assigned stratum
- RBC pre-treatment 24 h prior randomisation as documented in the eCRF
- Administration of fluids 24 h prior randomisation (yes/no)
- APACHE II total score at baseline (≤ 20 points, 21-30 points, >30 points)
- SOFA total score at baseline (< 10 points, ≥10 points)
- SOFA renal score at baseline (1-2, 3-5); score 1 = score 0 recorded in the eCRF (corresponding to creatinine < 110 µmol/L [1.2 mg/dL] or urine output ≥ 500 mL/day); score 2 = score 1 as recorded in the eCRF and so forth
- Administration of blood products during the study (yes/no)
- Septic shock / severe sepsis diagnosis at baseline (yes, no)
- Time of diagnosis of severe sepsis/septic shock (at ICU admission, during ICU stay)
- Occurrences of severe sepsis / septic shock (one occurrence, at least 2 occurrences) during study (as recorded in the database)
- Concomitant medication (patients who received only allowed concomitant medication, patients who received at least one dose of not allowed medication during study)
- Use of RRT during study (yes/no)
- Type of patient (trauma, surgical, medical)

If a subgroup included less than 10 patients, descriptive statistics were only presented for this subgroup category.
