# Supplementary material for: Efficacy and Safety of a Balanced Gelatine Solution for Fluid Resuscitation in Sepsis: A Prospective, Randomised, Controlled, Double-Blind Trial-GENIUS Trial
Source: J Clin Med. 2025 Jul 28;14(15):5323. doi: 10.3390/jcm14155323 (PMC12346933; doi:10.3390/jcm14155323)
Supplement: Supplementary file 1 [file jcm-14-05323-s001.zip › SDC5_Table S1_Time to Haemodynamic Stability by Subgroups.pdf]

Table S1. Time to haemodynamic stability by subgroups (ITT).

Subgroup	Statistics	Gelatin Group N =83	Crystalloid Group N = 84	p-value	
				Mann-Whitney Test	Van Elteren Test
Austria	N achieving HDS, n (%)	6 (100)	4 (66.7)		
	Time to HDS [hour]				
	Mean (SD)	1.7 (1.07)	1.0 (0.54)	0.2410	0.0609
Czech Republic	N achieving HDS, n (%)	9 (90.0)	9 (100)		
	Time to HDS [hour]				-
	Mean (SD)	2.5 (1.42)	2.8 (1.81)	1.0000	
France	N achieving HDS, n (%)	0	0		
	Time to HDS [hour]				
	Mean (SD)	-	-	-	-
Germany	N achieving HDS, n (%)	56 (94.9)	56 (91.8)		
	Time to HDS [hour]				
	Mean (SD)	5.5 (6.64)	7.0 (6.98)	0.1616	0.0701
Spain	N achieving HDS, n (%)	6 (75.0)	5 (83.3)		
	Time to HDS [hour]				
	Mean (SD)	3.4 (2.86)	2.0 (0.99)	0.5228	-
Site 11	N achieving HDS, n (%)	42 (97.7)	44 (95.7)		
	Time to HDS [hour]				
	Mean (SD)	5.1 (6.11)	7.3 (7.22)	0.0963	0.0911
Site 12	N achieving HDS, n (%)	3 (75.0)	5 (100)		
	Time to HDS [hour]				
	Mean (SD)	5.4 (4.45)	7.8 (9.57)	-	-
Site 13	N achieving HDS, n (%)	2 (100)	1 (100)		
	Time to HDS [hour]				
	Mean (SD)	18.0 (21.86)	4.3	-	-
Site 14	N achieving HDS, n (%)	3 (100)	3 (60.0)		
	Time to HDS [hour]				
	Mean (SD)	5.0 (4.08)	5.5 (2.92)	-	-
Site 15	N achieving HDS, n (%)	5 (83.3)	3 (75.0)		
	Time to HDS [hour]				
	Mean (SD)	4.2 (2.11)	4.4 (2.20)	-	-
Site 16	N achieving HDS, n (%)	1 (100)	0		
	Time to HDS [hour]				
	Mean (SD)	6.3	-	-	-
Site 21	N achieving HDS, n (%)	0	0		
	Time to HDS [hour]				
	Mean (SD)	0	-	-	-
Site 22	N achieving HDS, n (%)	0	0		
	Time to HDS [hour]				
	Mean (SD)	-	-	-	-
Site 41	N achieving HDS, n (%)	6 (100)	4 (66.7)		
	Time to HDS [hour]				
	Mean (SD)	1.7 (1.07)	1.0 (0.54)	0.2410	0.0609
Site 52	N achieving HDS, n (%)	9 (90.0)	9 (100)		
	Time to HDS [hour]				
	Mean (SD)	2.5 (1.42)	2.8 (1.81)	1.0000	-
Site 71	N achieving HDS, n (%)	4 (66.7)	5 (83.3)		
	Time to HDS [hour]				
	Mean (SD)	2.4 (1.48)	2.0 (0.99)	-	-
Site 73	N achieving HDS, n (%)	2 (100)	0		
	Time to HDS [hour]				

	Mean (SD)	5.4 (4.77)	-	-	-
RBC treatment 24h prior to randomisation (stratification factor)	N achieving HDS, n (%)<br>Time to HDS [hour]<br>Mean (SD)	15 (93.8)<br><br>3.6 (2.49)	17 (100)<br><br>5.4 (4.92)	0.4167	0.2090
No RBC treatment 24h prior to randomisation (stratification factor)	N achieving HDS, n (%)<br>Time to HDS [hour]<br>Mean (SD)	62 (92.5)<br><br>5.0 (6.43)	57 (85.1)<br><br>6.0 (6.88)	0.6114	0.4011
RBC therapy 24 h prior to randomisation (eCRF data)	N achieving HDS, n (%)<br>Time to HDS [hour]<br>Mean (SD)	13 (92.9)<br><br>3.9 (2.54)	14 (100)<br><br>6.1 (5.21)	0.4373	0.2136
Severe sepsis	N achieving HDS, n (%)<br>Time to HDS [hour]<br>Mean (SD)	20 (100)<br><br>4.0 (3.72)	16 (88.9)<br><br>5.2 (6.50)	0.8735	0.9215
Septic shock	N achieving HDS, n (%)<br>Time to HDS [hour]<br>Mean (SD)	57 (90.5)<br><br>4.9 (6.48)	58 (87.9)<br><br>6.0 (6.49)	0.4014	0.0837
sepsis/septic shock diagnosis - Diagnosis at ICU admission	N achieving HDS, n (%)<br>Time to HDS [hour]<br>Mean (SD)	9 (90.0)<br><br>3.9 (2.69)	11 (91.7)<br><br>2.8 (1.90)	0.4030	0.6298
sepsis/septic shock diagnosis - Diagnosis during ICU stay	N achieving HDS, n (%)<br>Time to HDS [hour]<br>Mean (SD)	68 (93.2)<br><br>4.8 (6.19)	63 (87.5)<br><br>6.4 (6.83)	0.1734	0.3159
Surgical patients	N achieving HDS, n (%)<br>Time to HDS [hour]<br>Mean (SD)	48 (92.3)<br><br>4.6 (5.46)	50 (87.7)<br><br>6.6 (6.69)	0.0762	0.0298
Trauma patients	N achieving HDS, n (%)<br>Time to HDS [hour]<br>Mean (SD)	8 (100)<br><br>3.9 (2.93)	5 (100)<br><br>3.8 (2.56)	0.8262	0.2386
Medical patients	N achieving HDS, n (%)<br>Time to HDS [hour]<br>Mean (SD)	21 (91.3)<br><br>5.2 (7.61)	19 (86.4)<br><br>4.3 (6.38)	0.1472	0.4507
Prior administration of fluids	N achieving HDS, n (%)<br>Time to HDS [hour]<br>Mean (SD)	73 (94.8)<br><br>4.8 (6.02)	69 (89.6)<br><br>5.8 (6.66)	0.5691	0.2975
No prior administration of fluids	N achieving HDS, n (%)<br>Time to HDS [hour]<br>Mean (SD)	4 (66.7)<br><br>3.3 (2.09)	5 (71.4)<br><br>5.9 (2.59)	-	-
Administration of blood products during study	N achieving HDS, n (%)<br>Time to HDS [hour]<br>Mean (SD)	48 (90.6)<br><br>5.6 (7.15)	45 (93.8)<br><br>7.0 (7.56)	0.3232	0.0597
No administration of blood products during study	N achieving HDS, n (%)<br>Time to HDS [hour]<br>Mean (SD)	29 (96.7)<br><br>3.1 (2.00)	29 (80.6)<br><br>4.0 (3.59)	0.9690	0.5556
RRT therapy during study	N achieving HDS, n (%)<br>Time to HDS [hour]<br>Mean (SD)	14 (73.7)<br><br>7.4 (9.25)	10 (83.3)<br><br>12.0 (10.07)	0.2081	0.3865
No RRT therapy during study	N achieving HDS, n (%)<br>Time to HDS [hour]<br>Mean (SD)	63 (98.4)<br><br>4.1 (4.74)	64 (88.9)<br><br>4.9 (5.19)	0.5307	0.4934
Occurrence of severe sepsis/septic	N achieving HDS, n (%)<br>Time to HDS [hour]	77 (92.8)	74 (88.1)		

shock - one occurrence at diagnosis	Mean (SD)	4.7 (5.88)	5.8 (6.46)	0.3716	0.1995
Occurrence of severe sepsis/septic shock - at least two occurrences (further occurrence after initial diagnosis)	N achieving HDS, n (%)<br>Time to HDS [hour]<br>Mean (SD)	11 (78.6)<br><br>4.3 (2.13)	14 (100)<br><br>8.2 (9.66)	1.0000	-
APACHE II at baseline ≤ 20	N achieving HDS, n (%)<br>Time to HDS [hour]<br>Mean (SD)	24 (96.0)<br><br>3.5 (3.87)	28 (93.3)<br><br>3.6 (3.21)	0.9561	0.7090
APACHE II at baseline 21-30	N achieving HDS, n (%)<br>Time to HDS [hour]<br>Mean (SD)	40 (93.0)<br><br>5.9 (7.36)	32 (88.9)<br><br>6.5 (7.01)	0.9413	0.6961
APACHE II at baseline > 30	N achieving HDS, n (%)<br>Time to HDS [hour]<br>Mean (SD)	8 (80.0)<br><br>3.0 (2.57)	9 (90.0)<br><br>8.1 (8.22)	0.0920	0.3711
APACHE II at baseline missing	N achieving HDS, n (%)<br>Time to HDS [hour]<br>Mean (SD)	5 (100)<br><br>3.7 (1.57)	5 (62.5)<br><br>10.1 (10.05)	0.0947	-
SOFA score at baseline < 10	N achieving HDS, n (%)<br>Time to HDS [hour]<br>Mean (SD)	53 (98.1)<br><br>4.6 (5.45)	48 (90.6)<br><br>4.9 (6.08)	0.6832	0.8302
SOFA at baseline ≥10	N achieving HDS, n (%)<br>Time to HDS [hour]<br>Mean (SD)	18 (78.3)<br><br>5.3 (7.87)	22 (88.0)<br><br>7.1 (6.51)	0.1655	0.6597
SOFA at baseline missing	N achieving HDS, n (%)<br>Time to HDS [hour]<br>Mean (SD)	6 (100)<br><br>3.3 (1.62)	4 (66.7)<br><br>10.3 (9.15)	0.0428	-
Using allowed concomitant medication only	N achieving HDS, n (%)<br>Time to HDS<br>Mean (SD)	64 (97.0)<br><br>4.5 (5.28)	59 (88.1)<br><br>5.3 (5.85)	0.4975	0.4085
Using any not allowed concomitant medication	N achieving HDS, n (%)<br>Time to HDS<br>Mean (SD)	13 (76.5)<br><br>5.7 (8.46)	15 (88.2)<br><br>7.8 (8.38)	0.6124	0.6945
RBC therapy 24 h prior to randomisation (eCRF data)	N achieving HDS, n (%)<br>Time to HDS [hour]<br>Mean (SD)	13 (92.9)<br><br>3.9 (2.54)	14 (100)<br><br>6.1 (5.21)	0.4373	0.2136
No RBC therapy 24 h prior to randomisation (eCRF data)	N achieving HDS, n (%)<br>Time to HDS [hour]<br>Mean (SD)	64 (92.8)<br><br>4.9 (6.35)	60 (85.7)<br><br>5.8 (6.75)	0.6030	0.4643
