# Supplementary material for: Efficacy and Safety of a Balanced Gelatine Solution for Fluid Resuscitation in Sepsis: A Prospective, Randomised, Controlled, Double-Blind Trial-GENIUS Trial
Source: J Clin Med. 2025 Jul 28;14(15):5323. doi: 10.3390/jcm14155323 (PMC12346933; doi:10.3390/jcm14155323)
Supplement: Supplementary file 1 [file jcm-14-05323-s001.zip › SDC6_Table S2_Clinical outcome parameter study group.pdf]

Table S2. Clinical outcome parameter for the overall study group (ITT).

Parameter	Statistics	Gelatine group N = 83	Crystalloid group N = 84	p-value*#
Duration of stay at ICU [days]	n	55	53	
	Mean (SD)	11.9 (9.32)	12.8 (10.98)	0.7958*
Duration of stay at hospital [days]	n	55	52	
	Mean (SD)	35.1 (22.99)	34.9 (20.68)	0.8934*
Patients who needed mechanical ventilation	n (%)	62 (74.7)	70 (83.3)	0.1705#
Patients with ongoing mechanical ventilation	n (%)	16 (19.3)	11 (13.1)	0.2779#
Cumulative days on invasive mechanical ventilation	n	62	70	
	Mean (SD)	12.4 (10.62)	10.0 (8.84)	0.4534*
Ventilator free days	n	62	70	
	Mean (SD)	4.2 (5.81)	3.6 (5.09)	0.5995*
RRT therapy	n (%)	19 (22.9)	12 (14.3)	0.1527#
Cumulative days on RRT therapy	Mean (SD)	15.3 (10.45)	9.3 (10.01)	0.1926*
RRT therapy ongoing at study end	n (%)	4 (4.8)	6 (7.1)	n/a
RRT therapy ongoing at study end (alive patients)	n (%)	0	0	n/a
New kidney disease occurred (FU)	n (%)	3 (3.6)	3 (3.6)	0.9519#
RRT since study termination	n (%)			
Yes		0	2 (2.4)	
No		64 (77.1)	59 (70.2)	0.1442#
Infection free days	n	80	76	
	Mean (SD)	4.3 (5.28)	4.4 (5.33)	0.6009*
Antibiotic free days	n	83	84	
	Mean (SD)	2.5 (4.39)	2.8 (4.81)	0.8903*
Vasopressor free days	n	58	55	
	Mean (SD)	9.9 (8.56)	8.7 (7.80)	0.4693*
Patients with at least one concomitant vasopressor treatment	n (%)	58 (69.9)	55 (65.5)	n/a
Norepinephrine		58 (69.9)	53 (63.1)	n/a
Vasopressin		8 (9.6)	1 (1.2)	n/a
Argipressin		1 (1.2)	1 (1.2)	n/a
Epinephrine		1 (1.2)	5 (6.0)	n/a
Desmopressin		0	1 (1.2)	n/a
Total number of treatments		698	827	n/a
Patients with at least one concomitant inotropic treatment	n (%)	19 (22.9)	18 (21.4)	n/a
Dobutamine		14 (16.8)	12 (14.3)	n/a
Milrinone		4 (4.8)	5 (6.0)	n/a
Digitoxin		1 (1.2)	0	n/a
Sildenafil		1 (1.2)	0	n/a
Epinephrine		0	2 (2.4)	n/a
Total number of treatments		52	67	n/a

Total number of new severe sepsis/septic shock diagnosis (further occurrence after initial diagnosis)	n	14	14	
	Mean (SD)	2.9 (6.67)	2.0 (2.15)	0.2383*
*Mann-Whitney-U test p-value (two-sided); #Chi-square p-value (two-sided)				
