# Supplementary material for: Efficacy and Safety of a Balanced Gelatine Solution for Fluid Resuscitation in Sepsis: A Prospective, Randomised, Controlled, Double-Blind Trial-GENIUS Trial
Source: J Clin Med. 2025 Jul 28;14(15):5323. doi: 10.3390/jcm14155323 (PMC12346933; doi:10.3390/jcm14155323)
Supplement: Supplementary file 1 [file jcm-14-05323-s001.zip › SDC7_Table S3_Shift table of KDIGO stages over study period.pdf]

**Table S3.** Shift table of KDIGO stages over study period (SAF). KDIGO=Kidney Disease Improving Global Outcome. KDIGO scores were calculated for all patients according to SAP derivation rules.

| Timepoint | Treatment | Stage at Baseline | Stage post Baseline | | | | |
|---|---|---|---|---|---|---|---|
| | | | Stage 0 n (%) | Stage 1 n (%) | Stage 2 n (%) | Stage 3 n (%) | Total n (%) |
| First Morning | Gelatin Group (N=83) | Stage 0 | 35 (77.8) | 4 (25.0) | 1 (10.0) | 1 (8.3) | 41 (49.4) |
| | | Stage 1 | 9 (20.0) | 9 (56.3) | 3 (30.0) | 4 (33.3) | 25 (30.1) |
| | | Stage 2 | 1 (2.2) | 3 (18.8) | 5 (50.0) | 3 (25.0) | 12 (14.5) |
| | | Stage 3 | 0 | 0 | 1 (10.0 | 4 (33.3) | 5 (6.0) |
| | | Total | 45 (100) | 16 (100) | 10 (100) | 12 (100) | 83 (100) |
| | Crystalloid Group (N=84) | Stage 0 | 46 (88.5) | 3 (25.0) | 0 | 0 | 49 (60.5) |
| | | Stage 1 | 6 (11.5) | 6 (50.0) | 1 (12.5) | 2 (22.2) | 15 (18.5) |
| | | Stage 2 | 0 | 3 (25.0) | 6 (75.0) | 3 (33.3) | 12 (14.8) |
| | | Stage 3 | 0 | 0 | 1 (12.5) | 4 (44.4) | 5 (6.2) |
| | | Total | 52 (100) | 12 (100) | 8 (100) | 9 (100) | 81 (100) |
| | Total (N=167) | Stage 0 | 81 (83.5) | 7 (25.0) | 1 (5.6) | 1 (4.8) | 90 (54.9) |
| | | Stage 1 | 15 (15.5) | 15 (53.6) | 4 (22.2) | 6 (28.6) | 40 (24.4) |
| | | Stage 2 | 1 (1.0) | 6 (21.4) | 11 (61.1) | 6 (28.6) | 24 (14.6) |
| | | Stage 3 | 0 | 0 | 2 (11.1) | 8 (38.1) | 10 (6.1) |
| | | Total | 97 (100) | 28 (100) | 18 (100) | 21 (100) | 164 (100) |
| Second Morning | Gelatin Group (N=83) | Stage 0 | 31 (77.5) | 4 (22.2) | 2 (22.2) | 1 (8.3) | 38 (48.1) |
| | | Stage 1 | 7 (17.5) | 11 (61.1) | 2 (22.2) | 5 (41.7) | 25 (31.6) |
| | | Stage 2 | 2 (5.0) | 2 (11.1) | 4 (44.4) | 3 (25.0) | 11 (13.9) |
| | | Stage 3 | 0 | 1 (5.6) | 1 (11.1) | 3 (25.0) | 5 (6.3) |
| | | Total | 40 (100) | 18 (100) | 9 (100) | 12 (100) | 79 (100) |
| | Crystalloid Group (N=84) | Stage 0 | 43 (87.8) | 4 (30.8) | 0 | 0 | 47 (61.0) |
| | | Stage 1 | 6 (12.2) | 5 (38.5) | 1 (14.3) | 2 (25.0) | 14 (18.2) |
| | | Stage 2 | 0 | 3 (23.1) | 5 (71.4) | 3 (37.5) | 11 (14.3) |
| | | Stage 3 | 0 | 1 (7.7) | 1 (14.3) | 3 (37.5) | 5 (6.5) |
| | | Total | 49 (100) | 13 (100) | 7 (100) | 8 (100) | 77 (100) |
| | Total (N=167) | Stage 0 | 74 (83.1) | 8 (25.8) | 2 (12.5) | 1 (5.0) | 85 (54.5) |

| | | | | | | | |
|---|---|---|---|---|---|---|---|
| | | Stage 1 | 13 (14.6) | 16 (51.6) | 3 (18.8) | 7 (35.0) | 39 (25.0) |
| | | Stage 2 | 2 (2.2) | 5 (16.1) | 9 (56.3) | 6 (30.0) | 22 (14.1) |
| | | Stage 3 | 0 | 2 (6.5) | 2 (12.5) | 6 (30.0) | 10 (6.4) |
| | | Total | 89 (100) | 31 (100) | 16 (100) | 20 (100) | 156 (100) |
| Day 3 | Gelatin Group (N=83) | Stage 0 | 34 (69.4) | 1 (16.7) | 2 (25.0) | 2 (12.5) | 39 (49.4) |
| | | Stage 1 | 11 (22.4) | 4 (66.7) | 4 (50.0) | 6 (37.5) | 25 (31.6) |
| | | Stage 2 | 4 (8.2) | 0 | 2 (25.0) | 4 (25.0) | 10 (12.7) |
| | | Stage 3 | 0 | 1 (16.7) | 0 | 4 (25.0) | 5 (6.3) |
| | | Total | 49 (100) | 6 (100) | 8 (100) | 16 (100) | 79 (100) |
| | Crystalloid Group (N=84) | Stage 0 | 46 (83.6) | 2 (20.0) | 0 | 0 | 48 (61.5) |
| | | Stage 1 | 7 (12.7) | 4 (40.0) | 1 (25.0) | 2 (22.2) | 14 (17.9) |
| | | Stage 2 | 1 (1.8) | 3 (30.0) | 3 (75.0) | 4 (44.4) | 11 (14.1) |
| | | Stage 3 | 1 (1.8) | 1 (10.0) | 0 | 3 (33.3) | 5 (6.4) |
| | | Total | 55 (100) | 10 (100) | 4 (100) | 9 (100) | 78 (100) |
| | Total (N=167) | Stage 0 | 80 (76.9) | 3 (18.8) | 2 (16.7) | 2 (8.0) | 87 (55.4) |
| | | Stage 1 | 18 (17.3) | 8 (50.0) | 5 (41.7) | 8 (32.0) | 39 (24.8) |
| | | Stage 2 | 5 (4.8) | 3 (18.8) | 5 (41.7) | 8 (32.0) | 21 (13.4) |
| | | Stage 3 | 1 (1.0) | 2 (12.5) | 0 | 7 (28.0) | 10 (6.4) |
| | | Total | 104 (100) | 16 (100) | 12 (100) | 25 (100) | 157 (100) |
| Day 4 | Gelatin Group (N=83) | Stage 0 | 28 (71.8) | 1 (16.7) | 0 | 3 (18.8) | 32 (50.0) |
| | | Stage 1 | 8 (20.5) | 3 (50.0) | 2 (66.7) | 7 (43.8) | 20 (31.3) |
| | | Stage 2 | 2 (5.1) | 0 | 1 (33.3) | 4 (25.0) | 7 (10.9) |
| | | Stage 3 | 1 (2.6) | 2 (33.3) | 0 | 2 (12.5) | 5 (7.8) |
| | | Stage | 39 (100) | 6 (100) | 3 (100) | 16 (100) | 64 (100) |
| | Crystalloid Group (N=84) | Stage 0 | 37 (84.1) | 0 | 0 | 1 (11.1) | 38 (59.4) |
| | | Stage 1 | 6 (13.6) | 2 (25.0) | 2 (66.7) | 2 (22.2) | 12 (18.8) |
| | | Stage 2 | 0 | 5 (62.5) | 1 (33.3) | 4 (44.4) | 10 (15.6) |
| | | Stage 3 | 1 (2.3) | 1 (12.5) | 0 | 2 (22.2) | 4 (6.3) |
| | | Total | 44 (100) | 8 (100) | 3 (100) | 9 (100) | 64 (100) |
| | Total (N=167) | Stage 0 | 65 (78.3) | 1 (7.1) | 0 | 4 (16.0) | 70 (54.7) |
| | | Stage 1 | 14 (16.9) | 5 (37.5) | 4 (66.7) | 9 (36.0) | 32 (25.0) |
| | | Stage 2 | 2 (2.4) | 5 (37.5) | 2 (33.3) | 8 (32.0) | 17 (13.3) |
| | | Stage 3 | 2 (2.4) | 3 (21.4) | 0 | 4 (16.0) | 9 (7.0) |

| | | | | | | | |
|---|---|---|---|---|---|---|---|
| | | Stage | 83 (100) | 14 (100) | 6 (100) | 25 (100) | 128 (100) |
| Day 5 | Gelatin Group (N=83) | Stage 0 | 27 (75.0) | 1 (33.3) | 0 | 3 (20.0) | 31 (51.7) |
| | | Stage 1 | 7 (19.4) | 2 (66.7) | 3 (50.0) | 7 (46.7) | 19 (31.7) |
| | | Stage 2 | 1 (2.8) | 0 | 2 (33.3) | 3 (20.0) | 6 (10.0) |
| | | Stage 3 | 1 (2.8) | 0 | 1 (16.7) | 2 (13.3) | 4 (6.7) |
| | | Total | 36 (100) | 3 (100) | 6 (100) | 15 (100) | 60 (100) |
| | Crystalloid Group (N=84) | Stage 0 | 34 (77.3) | 0 | 0 | 1 (14.3) | 35 (58.3) |
| | | Stage 1 | 6 (13.6) | 3 (42.9) | 1 (50.0) | 2 (28.6) | 12 (20.0) |
| | | Stage 2 | 3 (6.8) | 3 (42.9) | 1 (50.0) | 3 (42.9) | 10 (16.7) |
| | | Stage 3 | 1 (2.3) | 1 (14.3) | 0 | 1 (14.3) | 3 (5.0) |
| | | Total | 44 (100) | 7 (100) | 2 (100) | 7 (100) | 60(100) |
| | Total (N=167) | Stage 0 | 61 (76.3) | 1 (10.0) | 0 | 4 (18.2) | 66 (55.5) |
| | | Stage 1 | 13 (16.3) | 5 (50.0) | 4 (50.0) | 9 (40.9) | 31 (25.8) |
| | | Stage 2 | 4 (5.0) | 3 (30.0) | 3 (37.3) | 6 (27.3) | 16 (13.3) |
| | | Stage 3 | 2 (2.5) | 1 (10.0) | 1 (12.5) | 3 (13.6) | 7 (5.8) |
| | | Total | 80 (100) | 10 (100) | 8 (100) | 22 (100) | 120 (100) |
| Day 6 | Gelatin Group (N=83) | Stage 0 | 21 (70.0) | 1 (50.0) | 0 | 3 (21.4) | 25 (50.0) |
| | | Stage 1 | 7 (23.3) | 1 (50.0) | 2 (50.0) | 7 (50.0) | 17 (34.0) |
| | | Stage 2 | 1 (3.3) | 0 | 2 (50.0) | 3 (21.4) | 6 (12.0) |
| | | Stage 3 | 1 (3.3) | 0 | 0 | 1 (7.1) | 2 (4.0) |
| | | Total | 30 (100) | 2 (100) | 4 (100) | 14 (100) | 50 (100) |
| | Crystalloid Group (N=84) | Stage 0 | 32 (78.0) | 0 | 0 | 0 | 32 (59.3) |
| | | Stage 1 | 7 (17.1) | 4 (44.4) | 0 | 1 (25.0) | 12 (22.2) |
| | | Stage 2 | 2 (4.9) | 3 (33.3) | 0 | 3 (75.0) | 8 (14.8) |
| | | Stage 3 | 0 | 2 (22.2) | 0 | 0 | 2 (3.7) |
| | | Total | 41 (100) | 9 (100) | 0 | 4 (100) | 54 (100) |
| | Total (N=167) | Stage 0 | 53 (74.6) | 1 (9.1) | 0 | 3 (16.7) | 57 (54.8) |
| | | Stage 1 | 14 (19.7) | 5 (45.5) | 2 (50.0) | 8 (44.4) | 29 (27.9) |
| | | Stage 2 | 3 (4.2) | 3 (27.3) | 2 (50.0) | 6 (33.3) | 14 (13.5) |
| | | Stage 3 | 1 (1.4) | 2 (18.2) | 0 | 1 (5.6) | 4 (3.8) |
| | | Total | 71 (100) | 11 (100) | 4 (100) | 18 (100) | 104 (100) |
| Day 7 | Gelatin Group (N=83) | Stage 0 | 19 (70.4) | 0 | 1 (20.0) | 4 (30.8) | 24 (51.1) |
| | | Stage 1 | 7 (25.9) | 2 (100) | 2 (40.0) | 5 (38.5) | 16 (34.0) |

| | | | | | | | |
|---|---|---|---|---|---|---|---|
| | | Stage 2 | 1 (3.7) | 0 | 2 (40.0) | 3 (23.1) | 6 (12.8) |
| | | Stage 3 | 0 | 0 | 0 | 1 (7.7) | 1 (2.1) |
| | | Total | 27 (100) | 2 (100) | 5 (100) | 13 (100) | 47 (100) |
| | Crystalloid Group (N=84) | Stage 0 | 28 (75.7) | 0 | 0 | 1 (25.0) | 29 (60.4) |
| | | Stage 1 | 7 (18.9) | 3 (42.9) | 0 | 1 (25.0) | 11 (22.9) |
| | | Stage 2 | 1 (2.7) | 3 (42.9) | 0 | 2 (50.0) | 6 (12.5) |
| | | Stage 3 | 1 (2.7) | 1 (14.3) | 0 | 0 | 2 (4.2) |
| | | Total | 37 (100) | 7 (100) | 0 | 4 (100) | 48 (100) |
| | Total (N=167) | Stage 0 | 47 (73.4) | 0 | 1 (20.0) | 5 (29.4) | 53 (55.8) |
| | | Stage 1 | 14 (21.9) | 5 (55.6) | 2 (40.0) | 6 (35.3) | 27 (28.4) |
| | | Stage 2 | 2 (3.1) | 3 (33.3) | 2 (40.0) | 5 (29.4) | 12 (12.6) |
| | | Stage 3 | 1 (1.6) | 1 (11.1) | 0 | 1 (5.9) | 3 (3.2) |
| | | Total | 64 (100) | 9 (100) | 5 (100) | 17 (100) | 95 (100) |
