# Supplementary material for: Efficacy and Safety of a Balanced Gelatine Solution for Fluid Resuscitation in Sepsis: A Prospective, Randomised, Controlled, Double-Blind Trial-GENIUS Trial
Source: J Clin Med. 2025 Jul 28;14(15):5323. doi: 10.3390/jcm14155323 (PMC12346933; doi:10.3390/jcm14155323)
Supplement: Supplementary file 1 [file jcm-14-05323-s001.zip › SDC8_Table S4_eGFR and urine output.pdf]

Table S4. eGFR at baseline, Day 1, 2, 3, 7 and Day 28 and urine output at baseline, 24 h and 48 h.

	Gelatine group (N=83)		Crystalloid group (N=84)	
Parameter<br>Visit	Value	Change from Baseline	Value	Change from Baseline
**eGFR (mL.min.1.73 $m2^{-1}$)**				
Baseline				
n with data	79		80	
n missing	4		4	
Mean	60.2		59.8	
SD	35.49		33.45	
Day 1				
n with data	82	78	80	78
n missing	1	5	1	3
Mean	50.3	-9.6	55.6	-5.9
SD	39.76	30.75	40.69	26.58
Day 2				
n with data	79	76	76	73
n missing	0	3	1	4
Mean	51.6	-7.83	60.9	-2.2
SD	41.96	34.19	43.75	29.05
Day 3				
n with data	70	68	65	64
n missing	9	11	13	14
Mean	54.8	-7.0	60.5	-0.9
SD	43.82	35.73	40.64	30.93
Day 7				
n with data	46	44	45	44
n missing	1	3	3	4
Mean	66.4	3.2	75.2	8.3
SD	47.35	42.16	42.35	34.35
Day 28				
n with data	17	16	10	9
n missing	0	1	0	1
Mean	81.5	24.3	71.5	6.5
SD	44.95	45.11	42.69	59.47
**urine output (ml)**				

Baseline				
n with data	83		82	
n missing	0		1	
Mean	93.5		79.5	
SD	196.27		211.45	
24 h				
n with data	83	83	80	80
n missing	0	0	1	1
Mean	538.6	445.1	549.7	468.2
SD	507.33	552.83	471.44	528.91
48 h				
n with data	75	75	75	75
n missing	3	3	2	2
Mean	697.3	597.4	745.2	658.7
SD	866.78	910.29	806.14	839.85
