# Supplementary material for: Efficacy and Safety of a Balanced Gelatine Solution for Fluid Resuscitation in Sepsis: A Prospective, Randomised, Controlled, Double-Blind Trial-GENIUS Trial
Source: J Clin Med. 2025 Jul 28;14(15):5323. doi: 10.3390/jcm14155323 (PMC12346933; doi:10.3390/jcm14155323)
Supplement: Supplementary file 1 [file jcm-14-05323-s001.zip › SDC9_Table S5_Summary of Adverse Reactions.pdf]

Table S5. Summary of adverse reactions by system organ class (SOC) and preferred term (PT) (SAF) N = total number of patients.

**Primary System Organ Class** / **Preferred Term**	**Gelatine Group N = 83**		**Crystalloid Group N = 84**		**Total N = 167**	
	**Events**	**Patients**	**Events**	**Patients**	**Events**	**Patients**
Adverse reactions	4	4 (4.8)	1	1 (1.2)	5	5 (3.0)
Metabolism and nutrition disorders	1	1 (1.2)	1	1 (1.2)	2	2 (1.2)
Hypernatraemia	0	0	1	1 (1.2)	1	1 (0.6)
Hypervolaemia	1	1 (1.2)	0	0	1	1 (0.6)
Blood and lymphatic system disorders	1	1 (1.2)	0	0	1	1 (0.6)
Anaemia	1	1 (1.2)	0	0	1	1 (0.6)
Investigations	1	1 (1.2)	0	0	1	1 (0.6)
Haemoglobin decreased	1	1 (1.2)	0	0	1	1 (0.6)
Renal and urinary disorders	1	1 (1.2)	0	0	1	1 (0.6)
Acute kidney injury	1	1 (1.2)	0	0	1	1 (0.6)
